# Endovascular thrombectomy and post-procedural headache

**DOI:** 10.1186/s10194-017-0719-0

**Published:** 2017-01-28

**Authors:** Sabrina Khan, Faisal Mohammad Amin, Markus Holtmannspötter, Klaus Hansen, Anna Maria Florescu, Zainab Fakhril-Din, Julie Falkenberg Petersen, Hashmat Ghanizada, Cenk Ayata, David Gaist, Messoud Ashina

**Affiliations:** 1 Danish Headache Center, Department of Neurology, Rigshospitalet, University of Copenhagen, Copenhagen, Denmark; 2 Department of Radiology, Rigshospitalet, University of Copenhagen, Copenhagen, Denmark; 3 Department of Neurology, Rigshospitalet, University of Copenhagen, Copenhagen, Denmark; 4 Stroke Service and Neuroscience Intensive Care Unit, Department of Neurology, Massachusetts General Hospital, Harvard Medical School, Charlestown, MA USA; 5 Department of Neurology, Odense University Hospital, Odense, Denmark; 6 Department of Clinical Research, University of Southern Denmark, Odense, Denmark

**Keywords:** EVT, Stroke, Risk, Complications, Migraine

## Abstract

**Background:**

We investigated the prevalence of post-procedural headache in patients who have undergone thrombectomy for ischemic stroke, and correlated history of migraine with risk of peri-procedural complications. A total of 314 patients underwent thrombectomy at the Danish National Hospital from January 2012 to December 2014. Eligible subjects were phone-interviewed using a purpose-developed semi-structured questionnaire according to the International Classification of Headache Disorders 3, beta version criteria.

**Findings:**

Among 96 eligible subjects, there was a significant decrease in migraine (*p* = 0.022) within the first 3 months after EVT compared to 1 year before treatment, which was further evident at interview time (on average 1.6 years after EVT, *p* = 0.013). A minority of patients experienced headaches for the first time within 3 months of their EVT (migraine 2, TTH 9), which persisted at interview time for subjects with migraine. Out of 12 subjects with peri-procedural complications, 2 had a history of migraine with aura.

**Conclusion:**

Thrombectomy leads to a significant decrease in previously known migraine, and new onset of headache in a small subset of patients. A history of migraine does not appear to predispose to peri-procedural complications.

**Electronic supplementary material:**

The online version of this article (doi:10.1186/s10194-017-0719-0) contains supplementary material, which is available to authorized users.

## Introduction

Endovascular thrombectomy is an established procedure used to treat ischemic stroke. The risk of post-procedural headache has received little attention. Studies on the interplay between headache and endovascular treatment (EVT) are contradictory [1–3]. It is also unknown whether peri-procedural vascular complications are more prevalent in subjects with migraine history, or how such complications may affect headache patterns after EVT. To elucidate potential bidirectional links between endovascular thrombectomy, procedure-related complications, and headache we retrospectively assessed headache characteristics in a cohort of patients with ischemic stroke who had undergone thrombectomy.

## Methods

We identified all patients who had undergone EVT for ischemic stroke at a tertiary referral center, Copenhagen, Denmark between January 2012 and December 2014. We assessed medical records of all identified cases (*n* = 314) and excluded those with: 1) procedures other than thrombectomy, and 2) aphasia, or no command of Danish or English.

Eligible patients were phone interviewed once, on average 1.6 years (range: 0.2–3.0) after EVT, using a purpose-developed semi-structured questionnaire. We collected information on headache status 1 year and 1 month before EVT and after EVT (3 months after and at time of interview).

All headaches were classified according to the International Classification of Headache Disorders 3, beta version [4].

Frequencies and percentages were calculated for categorical variables and medians and range for continuous variables. For comparisons of categorical data, we used the McNemar test. All *p*-values were two-sided and *p*-values below 0.05 were considered statistically significant. All analyses were performed using IBM® SPSS® Statistics version 23.

## Findings

In total, ninety-six patients were eligible and included in the study (Table 1). Twenty-five subjects (26%) reported lifetime history of migraine, 14 of these (15%) had migraine with aura, and 25 subjects (26%) reported lifetime history of tension-type headache (TTH). One year prior to thrombectomy, sixteen subjects (17%) reported migraine, 11 of these (12%) had migraine with aura, and 15 subjects (16%) reported TTH.

**Table 1:** Clinical and demographic data in 96 stroke patients

Characteristics	
Median age at EVT (years)	67 years (range: 28–90)
Males (n)	55
Right-handed (n)	87
Median height (cm)	174 cm (range: 152–194)
Median weight (kg)	75 kg (range: 45–130)
Smokers (n)	60
Median pack years^a^	20 years (range: 1–92)
History of hypertension (n)	38
Medication history prior to EVT (n)	
- Antithrombotic therapy^b^ - Antihypertensive therapy^c^	28^d^ 38^e^
Medication status at interview time (n)	
- Antithrombotic therapy^b^ - Antihypertensive therapy^c^	80 52
Thrombolysis treatment for stroke (n)	69^f^
Procedures performed (n)	
- Thrombectomy - Thrombectomy + stent - Thrombectomy + coil + Onyx	89 6 1
Median time from procedure to interview (years)	1.6 years (range: 0.2–3.0)

^a^Pack year = defined as 20 cigarettes smoked every day for one year

^b^Antithrombotic medication included clopidogrel (*n* = 5), acetylsalicylic acid (*n* = 17), warfarin (*n* = 5), rivaroxaban (*n* = 1) and dabigatran (*n* = 1)

^c^Antihypertensive medication included angiotensin-II-inhibitors (*n* = 8), beta-blockers (*n* = 20), ACE inhibitors (*n* = 10), and calcium-antagonists (*n* = 7)

^d^8 missing records

^e^7 missing records

^f^3 missing records

### Headache status before and after EVT

**Fig. 1:**
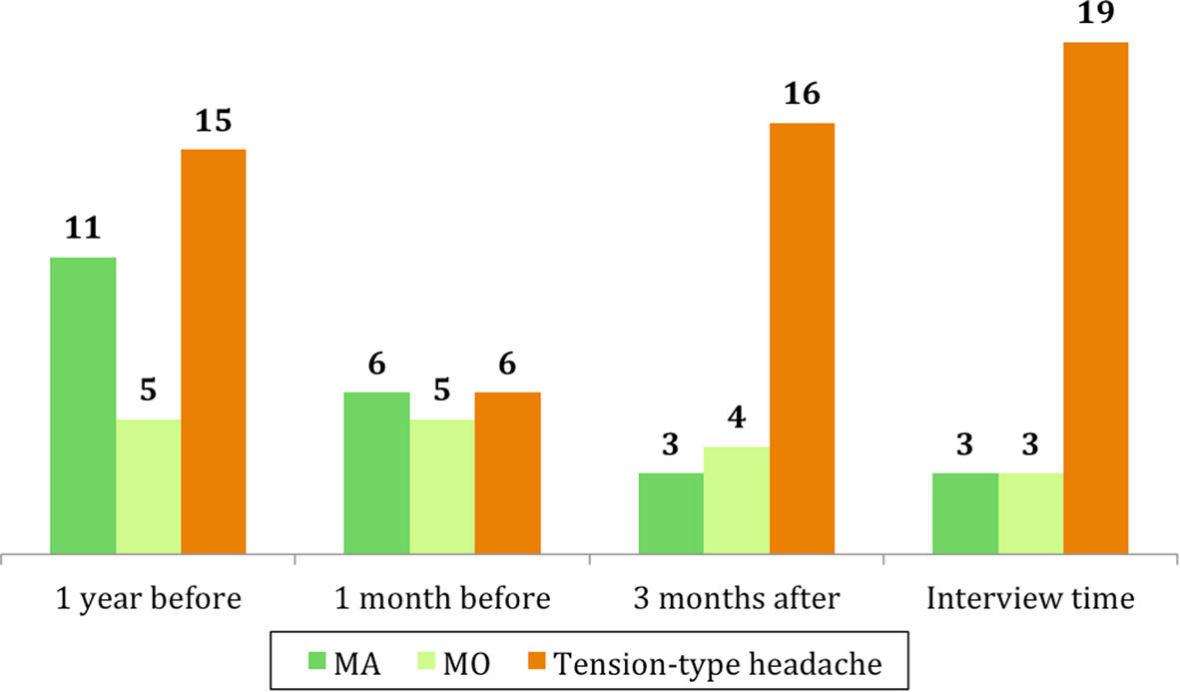
Headache status before and after thrombectomy. Numbers represent patients. Dotted line represents endovascular procedure. MA = migraine with aura, MO = migraine without aura

We found a significant decrease (56%) in migraine 3 months after thrombectomy compared to 1 year before the procedure (7 versus 16 subjects, *p* = 0.022) (Fig. 1, Additional file 1: Table S1). At “time of interview” (on average 1.6 years after EVT) we observed a larger (63%) reduction yet (6 versus 16 subjects, *p* = 0.013). For this time window, we saw no change in TTH.

We also compared patients’ headache status 1 month before thrombectomy with 3 months after treatment. Here we found an increase in TTH (6 versus 16 subjects, *p* = 0.013), which persisted at interview time (6 versus 19 subjects, *p* = 0.002). For this time window, we saw no change in migraine.

### De novo headaches after EVT

**Fig. 2:**
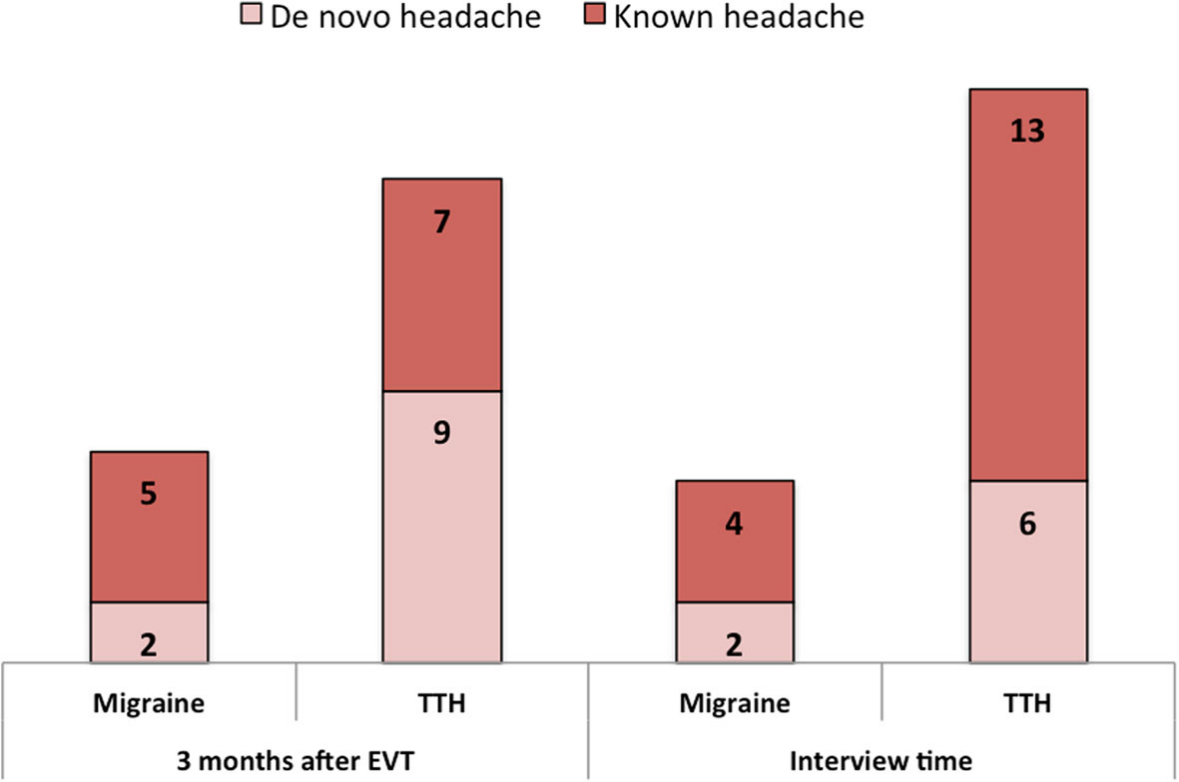
Distribution of de novo and known headache after thrombectomy. Numbers represent patients. TTH = tension-type headache

Nine subjects reported new onset of tension-type like headache during the first 3 months after EVT (6 episodic, 3 chronic) (Fig. 2). By time of interview, only 6 patients still experienced these new headaches (1 chronic, 4 episodic, 1 data not available).

Two female subjects experienced *de novo* migraine-like headache (one with and one without aura, ages 45 and 90 years) within the first 3 months after EVT, both reporting persistent headache at interview time, 2.1 and 1.3 years after EVT, respectively.

### Peri-procedural complications during EVT

In 12 of 96 subjects (12.5%), the thrombectomy procedure was complicated (Additional file 1: Figure S1 and Table S2). Peri-procedural complications arose in 8% of patients with a pre-EVT history of migraine (2 patients with migraine with aura) and 14% of patients with no history of migraine (*n* = 10).

## Discussion

Considering the focal intravascular manipulation inflicted on the vessels, headache after thrombectomy seems likely due to endothelial injury-induced inflammation of the intracranial vessel walls, activating sensory afferents. In support, a recent 3 T vessel wall MRI study reported contrast enhancement in arterial walls in patients treated with stent-retriever thrombectomy [5]. The bidirectional link between headache and EVT has thus far never been investigated, hence it is unknown whether history of headache may influence the endovascular procedure due to vascular complications such as distal embolization or dissection (Additional file 1: Table S2), and vice versa whether a complicated procedure may affect headache patterns after EVT. Studies on familial hemiplegic migraine type-1 mutant mice show larger infarcts and worse neurological outcomes after stroke compared to wild-type mice [6], specifically suggesting that a diagnosis of migraine with aura may predispose to vascular complications during the thrombectomy procedure.

As a major finding, we report a significant drop in migraine prevalence after thrombectomy for subjects with pre-EVT history of migraine, resulting in prevalence estimates close to those for the general population. We lacked a control group to assess for decline in migraine prevalence over time irrespective of EVT. However, we believe that the reduction of migraine observed in our study is beyond what one would expect from the natural history of this disorder [7]. This decrease could also be explained by the initiation of antithrombotic medication after thrombectomy [8]. No new prophylactic migraine treatment was initiated after EVT.

Contrary to migraine, we found that thrombectomy led to an increase in TTH after EVT, with a significantly larger prevalence compared to 1 month prior to treatment. However, when comparing the post-procedural prevalence with 1-year prior to thrombectomy, we observed no change, suggesting that thrombectomy does not result in added headache burden in the larger scope of life-time headache evolution. Differences in post-procedural medication (such as antithrombotic treatment) cannot explain this disparity between migraine and TTH, as all subjects were started on antithrombotic treatment after thrombectomy.

We also report new onset of headache in a subset of subjects (11%) who undergo thrombectomy. This headache resolves over time for some TTH cases, but persists for those reporting *de novo* migraine, suggesting that a fraction (8%) of subjects treated with thrombectomy may develop persistent new headache. Any *de novo* headache that occurs with close temporal relation to EVT is per definition a secondary headache, however, the current ICHD-3 beta criteria only allow any such headache to last up to 24 h [4]. This taxonomy is inadequate, as post-thrombectomy headache for the majority lasts at least 3 months, if not longer. Also, our results confirm the accepted notion that migraine *with* aura is overrepresented in a stroke cohort compared to the general population [9], and may even be larger in our cohort compared to other stroke case-based studies [10].

Finally, our results show that 12.5% of subjects undergoing thrombectomy experience peri-procedural complications. Albeit underpowered, these data suggest that migraine with aura does not increase the risk of complications during thrombectomy.

In conclusion, we have presented the first report of headache onset and prevalence after thrombectomy. Strengths of the study include a homogenous patient group, direct phone interviews, and ICHD-3 beta classification [5]. Limitations include recall bias and a relatively small sample size. Nevertheless, we believe the data help improve our understanding of post-procedural headache to optimize counseling of subjects who undergo thrombectomy with resultant headache. Larger, statistically robust prospective studies are warranted to establish the direction and magnitude of changes in migraine and other headaches after thrombectomy and to further explore migraine as a risk factor for peri-procedural complications.

## Additional file

Additional file 1: Table S1. Headache status before and after thrombectomy. Median time from endovascular treatment to interview: 1.6 years (range 0.2–3.0). **Table S2.** Classification system of peri-procedural complications. **Figure S1.** Peri-procedural complications in subjects with a life-time history of migraine. (DOCX 132 kb)
