# Supplementary material for: Endovascular thrombectomy and post-procedural headache
Source: J Headache Pain. 2017 Jan 28;18(1):10. doi: 10.1186/s10194-017-0719-0 (PMC5272848; doi:10.1186/s10194-017-0719-0)

**ONLINE SUPPLEMENT**

**Endovascular thrombectomy and post-procedural headache**

Sabrina Khan^1^, Faisal Mohammad Amin^1^, Markus Holtmannspötter^2^, Klaus Hansen^3^, Anna Maria Florescu^1^, Zainab Fakhril-Din^1^, Julie Falkenberg Petersen^1^, Hashmat Ghanizada^1^, Cenk Ayata^4^, David Gaist^5^, Messoud Ashina^1^

**Affiliations:**

*^1^ Danish Headache Center, Dept. of Neurology, ^2^ Dept. of Radiology, ^3^ Dept. of Neurology, Rigshospitalet, University of Copenhagen, Denmark*

*^4^Stroke Service and Neuroscience Intensive Care Unit, Department of Neurology, Massachusetts General Hospital, Harvard Medical School, Charlestown, MA, USA*

*^5^ Department of Neurology, Odense University Hospital, Denmark & Department of Clinical Research, University of Southern Denmark, Denmark*

**Cover title:** Post-procedural headache after thrombectomy

**Keywords:** EVT, stroke, risk, complications, migraine

**Corresponding author**

Prof. Messoud Ashina, M.D., Ph.D.
Tel: +45 38 63 30 54

E-mail: [ashina@dadlnet.dk](mailto:ashina@dadlnet.dk)

**Table S1** Headache status before and after thrombectomy. Median time from endovascular treatment to interview: 1.6 years (range 0.2-3.0)

|  | **1 year before (n)** | **3 months after (n)** | **Change (%)** | **P-value** |
| --- | --- | --- | --- | --- |
| Migraine | 16 | 7 | -56 | 0.022* |
| Tension-type headache | 15 | 16 | +6 | 1.000 |
|  | | | | |
|  | **1 year before (n)** | **Interview time (n)** | **Change (%)** | **P-value** |
| Migraine | 16 | 6 | -63 | 0.013* |
| Tension-type headache | 15 | 19 | +27 | 0.503 |
|  | | | | |
|  | **1 month before (n)** | **3 months after (n)** | **Change (%)** | **P-value** |
| Migraine | 11 | 7 | -36 | 0.388 |
| Tension-type headache | 6 | 16 | +167 | 0.013* |
|  | | | | |
|  | **1 month before (n)** | **Interview time (n)** | **Change (%)** | **P-value** |
| Migraine | 11 | 6 | -45 | 0.267 |
| Tension-type headache | 6 | 19 | +217 | 0.002* |

P values: difference between headache status before and after thrombectomy (calculated with the McNemar test).

**Table S2** Classification system of peri-procedural complications

| **Peri-procedural complications attributed to endovascular treatment**  **Classification criteria:** | |
| --- | --- |
| **1. Complication type** | a) Distal embolization |
|  | b) Rethrombosis |
|  | c) Dissection |
|  | d) Perforation |
|  | e) Technical* |
| **2. Degree of symptoms**** | a) Asymptomatic |
|  | b) Transient^a^ |
|  | c) Persistent^b^ |
| **3. Structural consequences***** | a) Ischemic |
|  | b) Hemorrhagic |
|  | c) Silent |

*Technical complications include stent occlusion and lack of vessel canalization.

**As determined from post EVT medical records

***As determined on CT scan 24 hours after EVT

^a^ Resolves within 24 hours of EVT

^b^ Lasts more than 24 hours after EVT

Medical records and CT scans were reviewed with the treating neurointerventionalist (MH) and neurologist (KH) to identify subjects where peri-procedural complications had occurred. Identified complications were classified as follows (n): distal embolization (7), rethrombosis (2), dissection (1), perforation (1), and technical complications (1). Of the 12 subjects with peri-procedural complications, 2 reported de novo tension-type like headache within the first 3 months after EVT, and 1 reported persistent headache at interview time.

**Figure S1** Peri-procedural complications in subjects with a life-time history of migraine


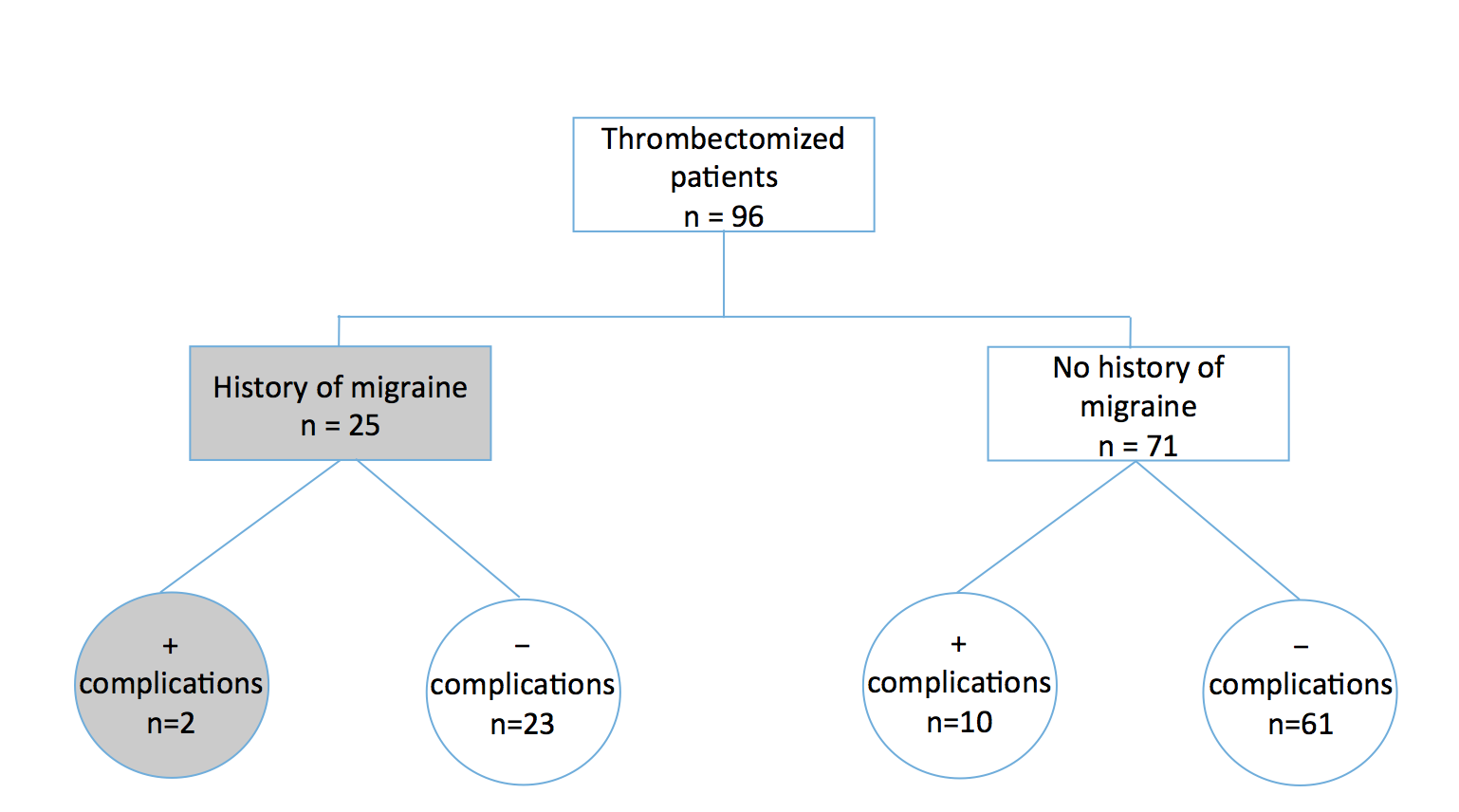

Supplement: Additional file 1: Table S1. — Headache status before and after thrombectomy. Median time from endovascular treatment to interview: 1.6 years (range 0.2–3.0). Table S2. Classification system of peri-procedural complications. Figure S1. Peri-procedural complications in subjects with a life-time history of migraine. (DOCX 132 kb) [file 10194_2017_719_MOESM1_ESM.docx]
